# The Advantage of Arriving First: Characteristic Times in Finite Size Populations of Error-Prone Replicators

**DOI:** 10.1371/journal.pone.0083142

**Published:** 2013-12-23

**Authors:** Arturo Marín, Héctor Tejero, Juan Carlos Nuño, Francisco Montero

**Affiliations:** 1 Departamento de Bioquímica y Biología Molecular I, Facultad de Ciencias Químicas, Universidad Complutense de Madrid, Madrid, Spain; 2 Departamento de Matemática Aplicada a los Recursos Naturales, Escuela Técnica Superior de Ingenieros de Montes, Universidad Politécnica de Madrid, Madrid, Spain; Manchester University, United Kingdom

## Abstract

We study the evolution of a finite size population formed by mutationally isolated lineages of error-prone replicators in a two-peak fitness landscape. Computer simulations are performed to gain a stochastic description of the system dynamics. More specifically, for different population sizes, we compute the probability of each lineage being selected in terms of their mutation rates and the amplification factors of the fittest phenotypes. We interpret the results as the compromise between the characteristic time a lineage takes to reach its fittest phenotype by crossing the neutral valley and the selective value of the sequences that form the lineages. A main conclusion is drawn: for finite population sizes, the survival probability of the lineage that arrives first to the fittest phenotype rises significantly.

## Introduction

The quasispecies model is a paradigm of the evolution of self-replicative sequences [1]–[3]. It assumes a population of error-prone replicators that evolves under the selective pressure caused by their competition under any constraint, e.g. constant population. Although this model has been mainly developed in the deterministic limit, i.e. under the assumption of infinite size population and fixed environmental conditions, the relevance of fluctuations in its dynamics was already stressed in Eigen's seminal paper [4]. Given a population of 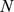 error-prone self-replicative sequences of 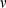 binary digits, the total number of different sequences that can be formed is 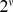. Therefore, the ratio 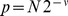 provides the probability of finding a particular sequence in a neutral landscape. This number is extremely low even for very large population sizes (e.g. if 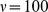 and 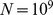, then 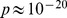). Fitness differences, together with initial conditions, make some sequences more frequent than others. Precisely, the fittest sequences, in the event that they exist, are the target of evolution by natural selection. Although the influence of the mutation rate in this evolutionary process has been widely studied [5]–[8], less attention has been paid to the relation of the mutation rate and the evolutionary time [9]–[11].

In the simplest non-neutral fitness landscape, it is assumed that all sequences except one, the master or fittest sequence, have equal fitness. If initially the population has a non-null proportion of the master sequence and the mutation rate is low enough, as time passes a distribution of mutant sequences is formed around the master sequence. This state is usually called quasispecies [4]. This distribution is quite stable even for finite size populations. On the contrary, as a consequence of the error-prone self-replication, the quasispecies can be destabilized if a higher second fitness peak (e.g. another sequence with a larger amplification factor) exists. The evolution towards the fittest sequence depends on several factors, mainly the mutation rate, the Hamming distance between the two peaks, the relative difference between the two fitness peaks and, as will be stressed in this paper, on the population size. For finite size populations, searching for new genotypes is restricted to a close neighborhood of the steady quasispecies. The exploration of the far distant sequence landscape is practically unreachable in finite time because, as has been said above, only populations of the order of 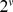 have a non-negligible probability of finding a new sequence located at a medium Hamming distance (e.g. 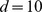).

Besides this limitation, finite size effects become apparent when competition between independent lineages occurs [12], [13]. If we consider two lineages formed by error-prone sequences that evolve in a two-peaks landscape, each with a different mutation rate, the question arises as to which of them will survive in the stationary state if initially each lineage occupies a fraction of the population. As we will see in the Results section, the answer depends on the size of the whole population. It is shown that optimal mutation rates exist that enhance the probability of survival of a lineage (and so, forming a quasispecies peaked around the fittest phenotype). Since having different mutation rates implies different evolutionary times, this result is explained as a consequence of arriving first to their fittest sequence.

In order to compute an evolutionary time in infinite populations described in terms of ordinary differential equations (e.g. using the molar fraction of each phenotype) the characteristic time has been introduced beforehand [14]. Recently, this approximation has been used to quantify the dependence of the evolutionary time on the mutation rate for different fitness landscapes [15]. We showed that, as a consequence of the trade-off between the searching capabilities and the fixation probabilities of the master sequences, the characteristic time exhibits a minimum for a positive mutation rate lower than the error threshold. We discussed the consequences of arriving first for a population of error-prone replicators and realized that having a low evolutionary time (i.e. a mutation rate close to the optimal value) could have determinant consequences in finite size populations. To evaluate the evolutionary time in this case we apply a generalization to this characteristic time [16]. However, its computation is seriously limited when either the population size or the mutation rate are too small.

The main goal of this paper is to study the evolution of a finite size population formed by mutationally isolated lineages of error-prone replicators in a two-peak fitness landscape and the influence that some parameters, namely the mutation rate and the population size, have on this dynamics. In all cases, competing lineages that have the same fitness landscape differ exclusively in their mutation rates. Since the final outcome of lineage competition will depend on the intra-lineage evolutionary processes, we first deal with this internal competition from a deterministic perspective in the first subsection of Results. Finite size effects in inter-lineage competition is studied computationally by means of a reduced model that is presented, justified and compared with the complete model in the next subsection in the Results. In the subsection “Lineage competition” this reduced model is applied to obtain the prevalence of each lineage in terms of their characteristic time and mutation rates for different population sizes. It is proven that the percentage of survival of the mutator lineages is not obviously dependent on the population size, which can be explained by the characteristic time of the lineages. In the Discussion we summarize our results and make some brief comments about their implications for real systems such as viruses and bacteria.

## Results

### Intra-lineage competition

Let us assume first a population of binary sequences of length 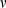 that forms a unique lineage. An amplification factor that measures its propensity to self-replicate is assigned to each sequence (its fitness). The model assumes a two-peak fitness landscape, i.e. there are three distinct phenotypes: 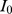, the sequence whose digits are all 0, 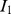, the sequence whose digits are all 1 and 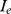, the error tail, that is formed by the rest of the sequences. The amplification factors of the master sequences 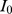 and 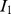 are 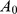 and 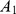, respectively. The amplification factor of the error tail is denoted as 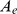 and verifies: 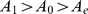. A similar fitness landscape with two equal peaks was previously applied in [17] to study the distribution of mutants in a degenerated quasispecies.

Self-replication is error-prone. As usual, 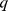 is the quality factor per digit, i.e. the probability of exact self-replication of each digit. The mutation rate 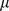 per digit is, therefore, 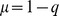. In reference to master sequence 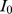, the sequences that differ in 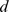 digits form the Hamming class 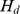. The mutation matrix that yields the probability that a sequence of Hamming class 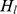 produces during replication a sequence of the Hamming class 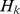 is given by [18].
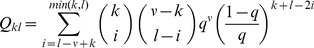
(1)If we assume that every sequence belonging to each Hamming class has the same amplification factor and that the total population is kept constant, the time evolution of the molar fraction of each Hamming class, 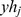 is described by the ODE system:
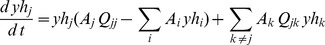
(2)for 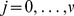. Here, without loss of generality, a null death rate of 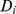 for all sequences has been assumed. Therefore, the selective value [4] of each Hamming class is 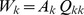.

If initially the whole population is considered to be formed only by master sequences 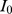 then, as time passes, a first quasispecies is obtained around 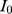 until it is displaced by the formation of a second quasispecies around the fittest genotype 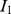. The latter quasispecies is asymptotically stable and its structure depends mainly on both the mutation rate 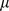 and the ratio 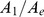.

**Figure 1:**
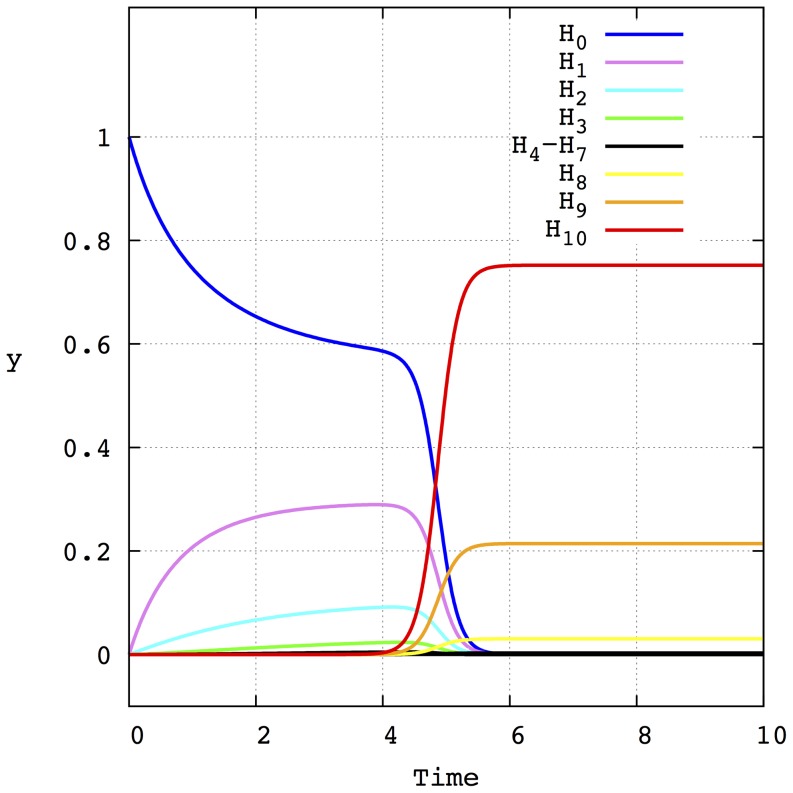
Time evolution of the molar fraction of each of the eleven Hamming classes (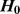 to 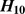) that form the sequence space when 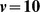. Initially, the whole population is formed by sequences 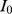, i.e. 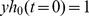. It is assumed that the amplification factors of all the sequences that belong to the Hamming classes 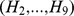 are equal and are given by 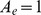. The amplification factors of the master copies that form the Hamming classes 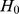 and 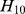 are 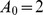 and 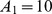, respectively. The mutation rate is 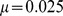.

Throughout the paper we will take a sequence length 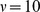. For this case, the non-linear ODE system of Eq. (2) has eleven differential equations that can be solved numerically using, for instance, a Runge-Kutta method implemented in MATLAB. The characteristic time for the time evolution of the master copy 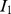, denoted as 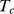, is then computed as in [14], [15] (see also the section Methods). As an example, Fig. 1 depicts the evolution of the molar fractions of each Hamming class for the initial value problem with 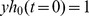 and 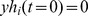 for all 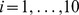 for a mutation rate 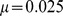. The amplification factors are taken: 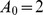, 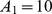 and 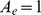. As can be seen in Fig. 1, the population of the different Hamming classes appears and disappears successively until a stationary state is achieved. This stationary state is formed by a distribution of Hamming classes around the fittest sequence 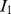, forming a quasispecies. Two important points are worth stressing here. The first one is that the mutation rate determines the characteristic time of the formation of this final quasispecies. The second one concerns the low concentration that the mutant phenotypes have during the evolution from the master phenotype 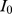 to the other master 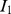. It is precisely the convergence of both factors that makes internal fluctuations especially relevant when several lineages with different mutation rates compete in finite size populations. Indeed, for finite size populations, having a lower characteristic time that allows them to reach the fittest phenotype first could favor the selection of the lineage with the larger mutation rate (contrary to the deterministic prediction) because the phenotypes of the other lineage die out. Obviously, in the limit of infinite population sizes having a low characteristic time is not relevant because, independently of the intermediate low concentration of the mutants the lineage with the lower mutation rate, which has a larger selective value, will asymptotically dominate the equilibrium population (forming a quasispecies around its fittest genotype 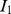). The influence of the characteristic time on the selective properties of independent lineages will be explored in detail in the following sections.

However, first at all, we have to overcome a technical problem caused by the natural computational limitations. The model presented in the previous paragraphs assumes a certain Hamming distance between the two master sequences. In the deterministic limit, this distance can be covered in a reasonable time since an infinite number of sequences are self-replicating and, as a consequence of mutation, effectively looking for new genotypes in the sequence space. However, when the size of the population is finite and much lower than the size of the sequence space 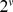, the searching capabilities of the population are drastically reduced and the computational time rises enormously. This fact, in practice, prevents the computation of the characteristic time and, consequently, a complete study of the finite size effects in the evolution of this kind of replicator systems.

#### A reduced model

To avoid this drawback, we define a reduced model that considers all the Hamming classes from 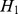 to 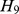 as only one class, 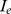, that can be taken as an error-tail of both master sequences. Essentially, this reduction rescales the evolutionary time of the population. In so doing, the complete ODE system of Eq. (2) of dimension 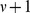 is simplified to the following tridimensional ODE system:
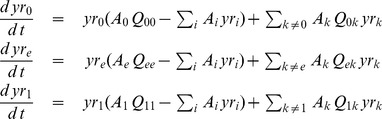
(3)where 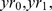 and 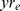 are the molar fractions of the master copies 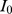, 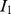 and the error tail 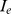 in the reduced scheme.

A reasonable choice for the mutation rate of the error-tail, 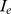, for either of the master copies, 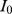 and 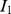, is as a weighted average rate over the intermediate Hamming classes, i.e.
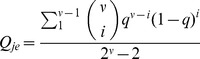
(4)for 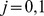. The combinatorial term takes into account the number of sequences that form each of the Hamming classes. Thus, the mutation matrix for this model is given by:
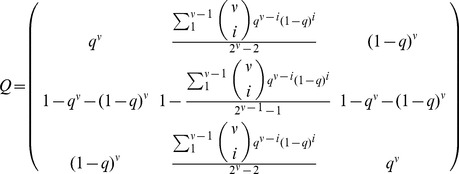
(5)


**Figure 2:**
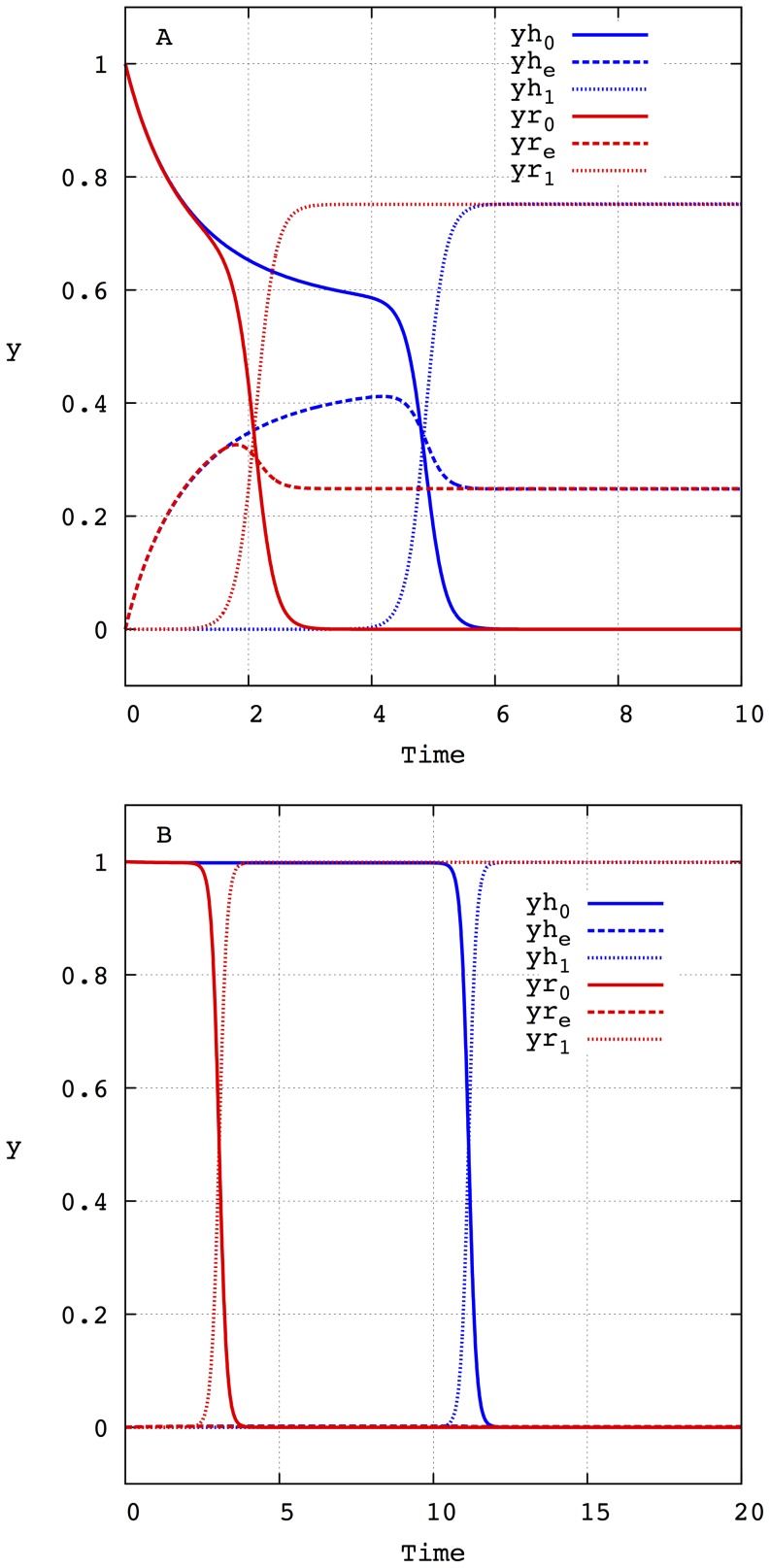
Time evolution of the molar fractions of each of the three phenotypes (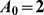, 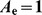 and 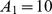) for the h-model divided into Hamming classes (denoted by the subindex 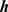 and curves in blue) and the reduced r-model (subindex 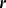 and red curves). The mutation rates are (A) 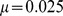 and (B) 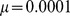. Note that the trajectories of both models are quite similar to the trajectories corresponding to the reduced model shifted to the left i.e. to lower values of time.

**Figure 3:**
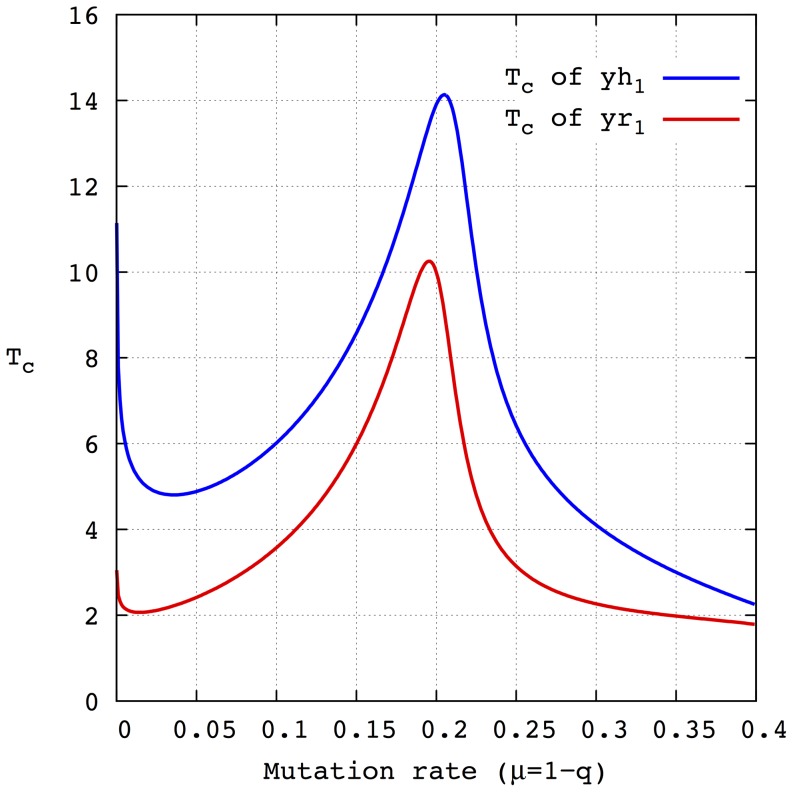
Characteristic time 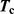 of the molar fraction of the phenotype 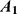 whose genotype is formed by all 1 as a function of the mutation rate for both the h-model (blue) and the r-model (red). The mutation rate varies in the interval 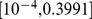 in constant steps of 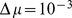. The amplification factors are 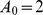, 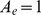 and 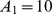. Note that both curves are qualitatively similar to that corresponding to the reduced model shifted to lower values of the characteristic time.

Fig. 2A and 2B compare the trajectories obtained by numerical integration of Eq. (3) and the complete system of Eq. (2) when 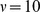 and 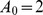, 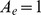 and 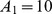 for two mutation rates 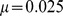 and 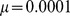, respectively. As can be seen, both systems behave similarly although over a different time scale. As expected, the same stationary state is reached but sooner in the reduced system. Fig. 3 depicts the characteristic time 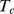 as a function of the mutation rate for both models for 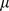-values in the interval 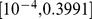 taken at steps of 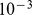. As before, the values of the amplification factors are 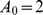, 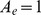 and 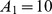. As can be appreciated in Fig. 3, the 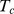-curve of the reduced model is qualitatively similar to that of the general model, though it is displaced to lower values. The differences between both models are more significative for low values of the mutation rate. Note that for mutation rates larger than 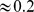 the population is in error catastrophe and the phenotype with the largest amplification factor is no longer selected [19]. In conclusion, at least at a qualitative level, the reduced model provides a reasonable description of the evolutionary behavior of the population but in a much shorter time scale. As will be shown in the next section, this reduction is going to allow an exhaustive study for low size populations.

**Figure 4:**
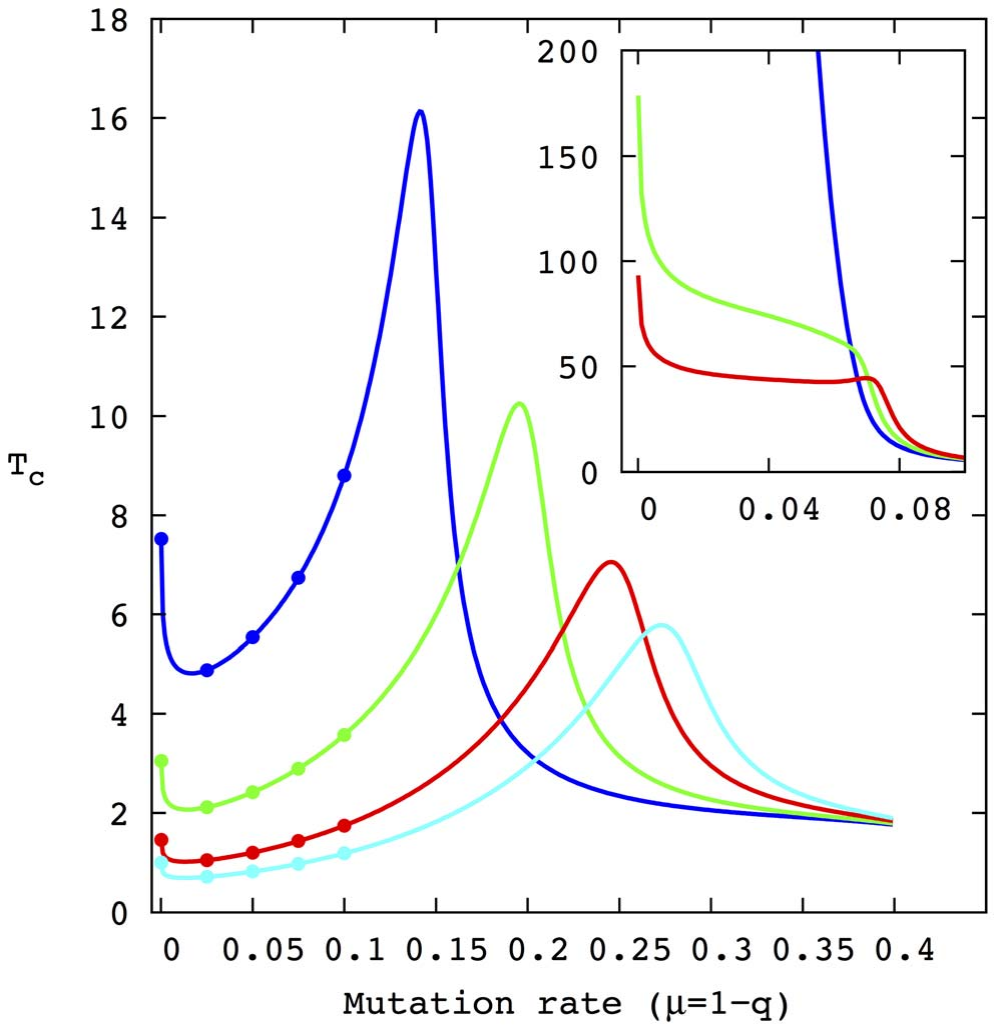
Values of the characteristic time for the reduced r-model for mutation rates in the range 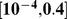 at constant steps 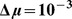. In the main figure the amplification factor 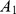 of the fittest phenotype is: 5 (blue curve), 10, (green), 20 (red) and 30 (cyan). In the inset, 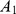 takes the values: 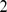 (blue curve), 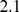 (green curve) and 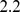 (red curve). In all cases, 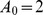 and 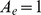. The points in the curves of the main picture correspond to the values mutation rate (from left to right), 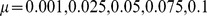. These values are applied later in stochastic simulations. As expected, increasing the value of the highest peak in the sequence landscape, 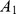, reduces the characteristic time. Furthermore, as depicted in the inset, as 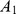 approaches 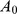 the curves tend to become monotonous and move up several order of magnitude.

The characteristic time of the time evolution of 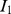 for different values of the amplification factor 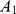 for the reduced model is shown in Fig. 4. The figure depicts 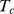 for 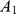 ( = 2, 2.1, 2.2, 5, 10, 20, and 30) as a function of the mutation rate 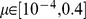 (with a 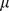-step equal to 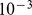). Since 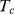 takes different scales as the value of 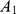 approaches that of 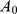, the curves for 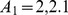 and 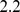 have been included in the inset. As before, 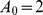 and 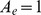. As it can be observed, the curves for large values of 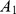 are qualitatively similar, all exhibiting a minimum value for approximately the same mutation rate 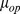 and a relative maximum near the error catastrophe (an extended description of this behavior has been previously presented in [15]). Note that as the amplification factor 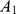 decreases, the curves move to the left and to higher values of 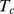. In the limit, when 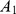 tends to 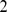 from above, the characteristic time increases enormously (several orders of magnitude higher than the scale used in Fig. 4) for all values of 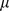. Moreover, the relative maxima disappear in the degenerate case 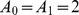, while the characteristic time reduces monotonously with 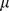 before entering the error catastrophe. The five points in the curves show the values that will be analyzed in more detail in the following subsections.

#### A stochastic simulation

As has already been stressed, the size of real populations is much lower than the size of the sequence space and, therefore, finite size effects may become relevant. If, in addition, competition is present, the deterministic approximation that considers infinite size populations does not assure reasonable results. Different approaches have been proposed to handle finite size populations [20]–[22]. In general, analytic methods that search for explicit solutions have practically been discarded due to the system complexity. Instead, computational algorithms have proven to be very efficient, although very time-consuming [23]. In this paper, we have used a Gillespie's stochastic simulation algorithm (SSA) [21] to carry out simulations of a finite size population of sequences that is described by the reduced model presented in the previous subsection Eq. (3).

To compute the characteristic time of the time evolution of the fittest sequence in each simulation first we average its population over the last 1000 steps and second, we compensate the areas over the averaged curve with those below the curve. This is equivalent to considering a straight line as the asymptotic value of the population [16]. All the simulations are run long enough to assure that the population has reached its asymptotic phase and that its average value is approximately that of the stationary state. Table 1 shows the average values of the characteristic times and the standard deviations generated from 200 independent simulations for five mutation rates and different population sizes. All the simulations have been performed using sequences of length 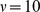 and amplification factors: 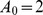 and 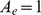. As before, initially the whole population is composed by master sequences 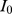. As can be seen, the characteristic time is very large for the lowest mutation rate, 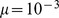 in comparison with the rest, mainly caused by the high searching time. Large characteristic times also appear for the largest mutation rate analyzed, 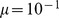. However, in this case, this is a consequence of the high values of the fixation time, i.e. once the fittest phenotype 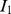 is found, the time the population takes to stabilize the quasispecies peaked around 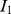. Between these two extremes, the stochastic 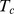 exhibits a minimum value that occurs, depending on the population size, in 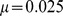 or 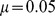 (as indicated by a superscript in the table).

**Table 1:** Mean and standard deviation of the characteristic time 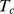 obtained in the stochastic simulations.

*A*_1_	*N*	Mutation rate				
		10^−3^	2.5×10^−2^	5×10^−2^	7.5×10^−2^	10^−1^
5	10^5^	90±83	5.1±0.4^a^	5.6±0.3	6.8±0.2	8.8±0.3
	10^6^	15±8	4.9±0.1^a^	5.54±0.08	6.74±0.07	8.8±0.1
	10^7^	8±2	4.88±0.04^a^	5.53±0.02	6.74±0.03	8.81±0.03
10	10^5^	80±81	2.4±0.3^a^	2.5±0.2	3±0.1	3.6±0.2
	10^6^	10±7	2.1±0.1^a^	2.42±0.06	2.9±0.05	3.57±0.04
	10^7^	5±2	2.12±0.03^a^	2.42±0.02	2.89±0.01	3.57±0.01
20	10^5^	58±58	1.3±0.3	1.3±0.2^a^	1.5±0.1	1.8±0.1
	10^6^	9±7	1.08±0.08^a^	1.21±0.04	1.43±0.04	1.75±0.03
	10^7^	3±1	1.05±0.02^a^	1.2±0.01	1.42±0.01	1.74±0.01
30	10^5^	58±54	1±0.3	0.9±0.1^a^	1±0.1	1.2±0.1
	10^6^	7±6	0.76±0.08^a^	0.83±0.05	0.98±0.04	1.19±0.03
	10^7^	3±1	0.71±0.02^a^	0.82±0.01	0.97±0.01	1.18±0.01

a Lowest values of 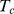.

Mean and standard deviation of 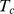 for the phenotype 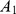 for different values of the mutation rate, population size and amplification factor 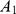. In all simulations, the whole population is initially formed by sequences 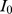 with an amplification factor 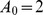. As before, the amplification factor of the error tail 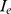 is 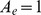. For each experimental setup 200 runs were performed.

### Lineages competition

Competition is another factor that can enhance finite size effects on populations of replicators. We postulate that the sequences of each lineage cannot change their mutation rate. This is a reasonable assumption when the mutation rate varies on a time scale greater than that of the competition [24]. Since lineages are independent of each other, the extinction of one lineage is an absorbing barrier. As a consequence, the internal noise inherent to finite size populations can completely change the fate of evolution. In this section, lineages with different mutation rates compete under a constant in average and finite total population constraint.

Let us first consider a population of two lineages, 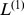 and 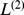, each one formed by three phenotypes 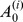, 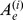 and 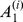 for 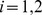 that evolve in a two-peaks landscape. The mutation rates per digit of the sequences in each lineage are denoted by 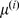 (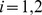). As before, the amplification factors of the sequences are 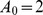, 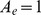 and 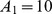 and equal for both lineages.

**Figure 5:**
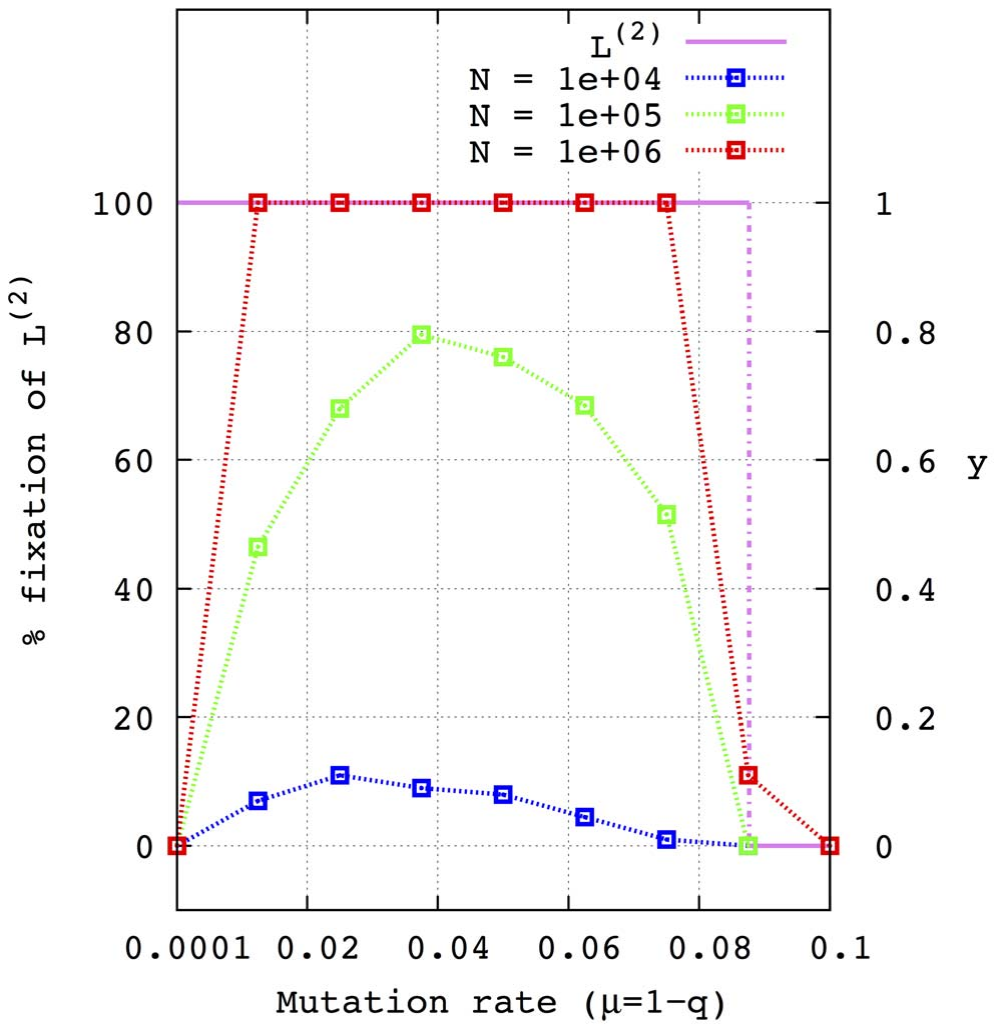
Percentage of fixation of lineage 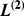 formed by error-prone self-replicative sequences against lineage 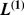 formed by a sequence with a null mutation rate (i.e. 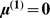) for different population sizes 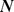. 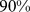
 of the initial population is formed by genotypes 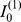 of lineage 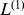 and the rest 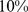 of genotypes 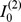. The amplification factors are: 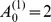 for 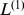 and 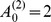, 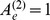 and 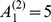 for 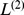. The mutation rate of the sequences of 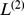, 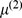, ranges from 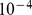 to 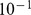. Concretely, the 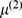-values used are: 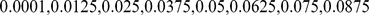 and 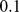. The population sizes that correspond to each curve are: 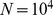 (blue line), 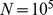 (green) and 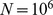 (red). The right vertical axis represents the deterministic molar fraction. The violet curve represents the equilibrium molar fraction obtained by numerical integration of the ODE system for values of 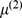 at constant steps of 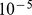. Note that in the deterministic limit of infinite population an abrupt transition occurs at a mutation rate of 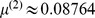. As it can be seen, this transition occurs gradually for finite size populations.

One interesting case occurs when one of the lineages is error free, i.e. 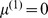. We want to estimate the probability of fixation of the other lineage for different mutation rates 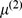. From the analysis of the deterministic equations, there exists a critical value 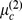 such that if 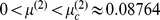, the lineage 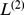 takes over the entire population independently of the initial conditions. Otherwise, 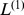 is selected for all the initial conditions. This result is no longer valid for finite size populations as is shown in Fig. 5. In fact, the probability of fixation of lineage 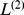 is less than 1, i.e. less than 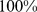 of the simulations yield a fixation of 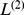 for all 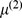 for 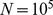. For 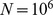, this probability equals 1 for some values of 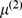 within an interval contained in 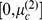. Note that, contrary to the infinite approximation, when 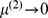 the probability of fixation also converges to 0. A smooth transition to null probability also appears for 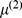-values below the deterministic critical value 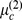. The results depicted in Fig. 5 are obtained from 200 simulations and an initial population divided into 90 per cent 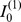 and 10 per cent 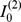.

**Figure 6:**
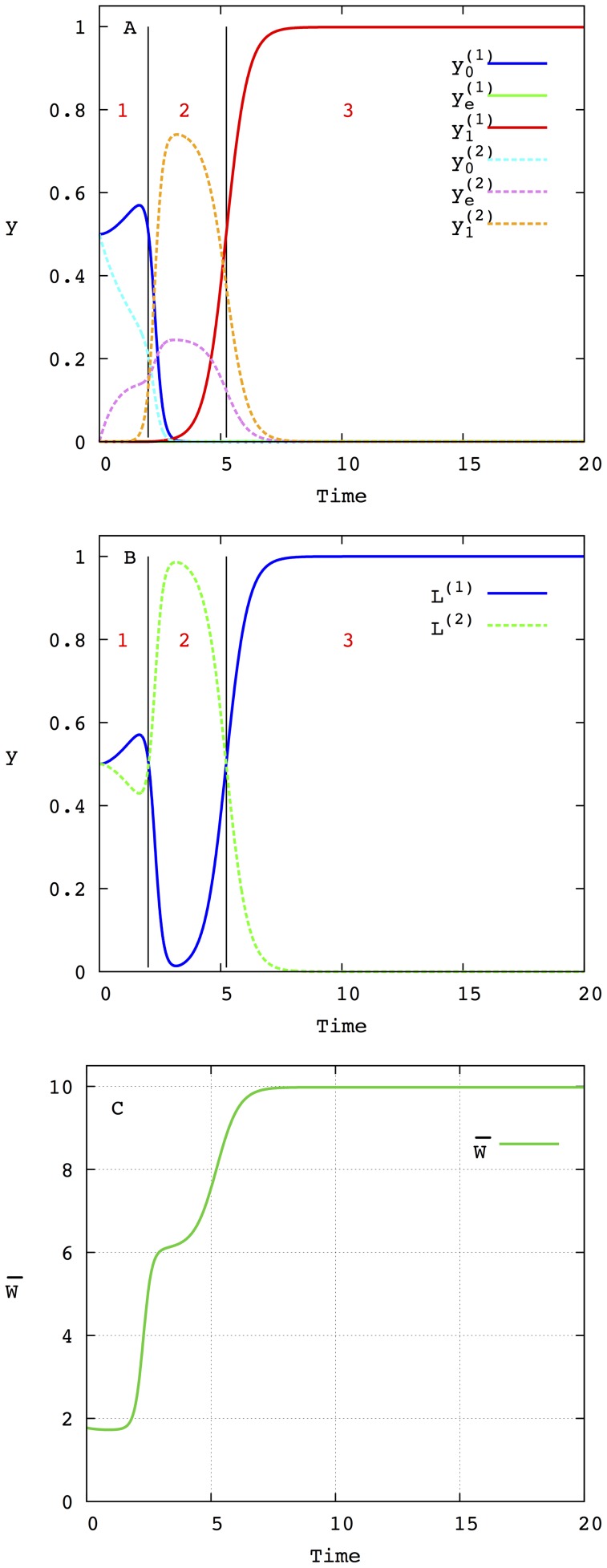
Lineage competition in the deterministic limit obtained by numerical integration of the corresponding ODE system. The mutation rates of the two lineages 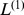 and 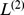 are 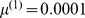 and 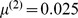, respectively. As before, the amplification factors of each phenotype in both lineages are: 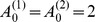, 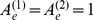 and 
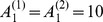
. The whole population is initially formed by genotypes 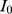, shared equally in both lineages. Figure (A) depicts the time evolution of each of the three phenotypes that form each lineage. Solid curves correspond to phenotypes of 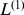, whereas dashed lines are for phenotypes of 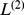. In figure (B) the three phenotypes are aggregated to yield the molar fraction of each lineage, 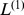 (solid blue line) and 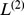 (dashed green line). In (C) the time evolution of the average fitness of the population (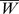) is shown. The three phases that appear in the temporal evolution of the phenotypes and molar fractions of the lineages are separated by vertical black lines (see main text for more details).

When the mutation rate of the sequences that form both lineages is larger than 0, concretely 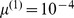 and 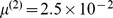, the dynamics become more complex. Fig. 6A shows the time evolution of the molar fraction of all the phenotypes 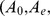 and 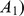 of each lineage in the deterministic approximation (obtained by numerical integration of the corresponding ODE systems Eq. (3)). The total molar fraction of each of the lineages is also shown in Fig. 6B. As can be seen, the system tends asymptotically to select the lineage with the largest selective value that corresponds to that with the lowest mutation rate, i.e. 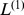. Nevertheless, the characteristic time of lineage 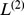 is small enough with respect to the corresponding 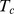 of lineage 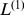 to give rise to three phases in the dynamics: (i) an increase in the proportion of 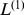 in the population, with the symmetric decrease of the proportion of 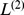. In this phase, none of the lineages have achieved their largest phenotype with 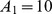. But, because the selective values for their master sequences with 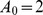 verify 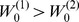, then 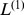 displaces 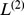, at least momentarily. (ii) Since the mutation rate of 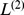 is much larger than that of 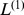, its characteristic time is much lower and its corresponding fittest sequence is found first. This phenotype self-replicates better than the rest and, in consequence, almost displaces lineage 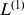 although, at this time, it is mostly formed by sequences 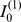 and 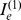. (iii) Finally, the lineage 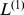 finds its best phenotype and, because 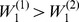, grows to reach its stationary concentration and displaces the phenotypes of lineage 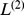 that becomes extinct. Consequently, after this third phase, the whole population is formed only by sequences of 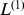. Importantly, the action of internal noise in the second phase of the time evolution of the lineages is going to be responsible for the disparity between the results obtained in the finite and infinite approximations. Finally, it is worth mentioning that the average selective value of the population 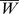 approaches asymptotically its maximum value 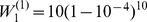, although it can decrease momentarily in some of the phases of evolution (e.g. during the first phase, in the case depicted in Fig. 6C).

**Figure 7:**
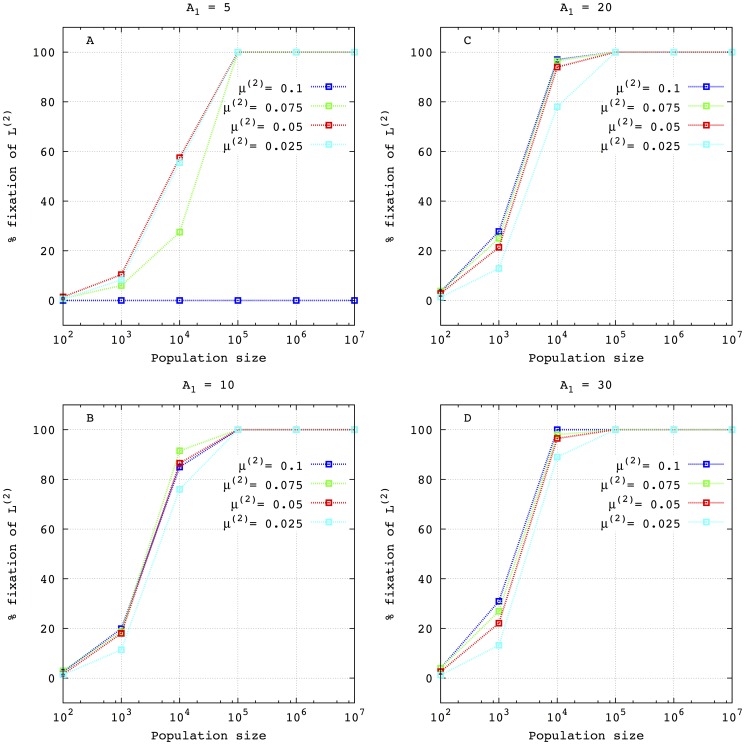
Percentage of fixation of lineage 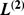 as a function of the population size in the competition against the other lineage 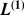 for different values of the mutation rate of the sequences that form 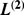 and for different values of the amplification factor 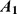: (A) 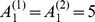; (B) 
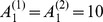
; (C) 
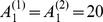
 and (D) 
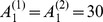
. In all cases, the values of the other amplification factors are 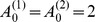 and 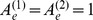 and the mutation rate of 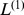 is 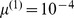. As before, the initial population is divided equally into genotypes 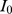 of both lineages. The values of 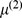 used are: 0.1 (blue lines), 0.075 (green lines), 0.05 (red lines) and 0.025 (cyan lines). The population sizes simulated are: 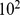, 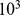, 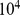, 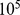, 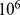 and 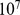. For low populations sizes 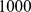 runs were carried out for each experimental setup, whereas for 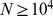 two hundred runs were enough to have negligible statistical errors.

Fig. 7 shows how the probability of fixation of lineage 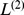 varies with the population size and the amplification factor of the fittest phenotype 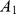. In all these experiments the mutation rate of lineage 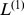 is 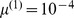. The rest of the parameters are kept as before, i.e. 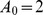, 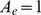. Initially, the two lineages are equally represented in the whole population and they are formed exclusively by phenotypes 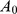. Each experiment has been repeated 1000 times for low population sizes and 200 times for larger ones. Fig. 7A shows the percentage of fixation of 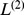 when 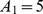. In this case, larger values of the mutation rate 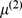 yield lower probabilities of fixation of 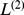. In Fig. 7B, when 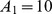, the results are not so evident, although seems to be opposite, i.e larger mutation rates 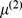 give rise to larger probabilities of fixation of 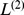. In the other two figures, 7C and 7D, the situation is clearly established. Besides, the probability of fixation of 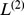 for all population sizes and all mutation rates shows a monotonous dependence on 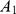. Note that, in contrast to the deterministic description, all curves converge to a 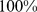 fixation for high population sizes. The exception is the case 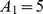 for the mutation rate 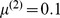 where the probability of fixation of lineage 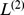 remains null for all population sizes. This result can be explained by the closeness of this mutation rate to the error threshold.

**Figure 8:**
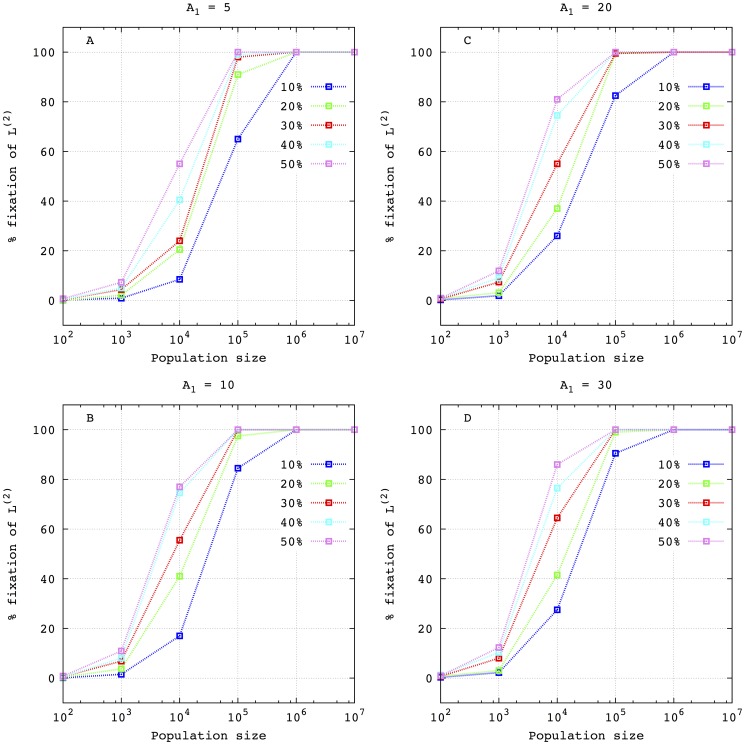
Percentage of fixation of lineage 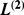 in terms of the population size in the competition against 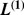 for different initial percentages of 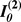: 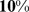 (blue lines), 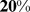 (green lines), 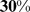 (red lines), 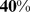 (cyan lines) and 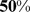 (violet lines). In all cases, the rest of the population is formed by 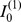. Each graph considers a different value of the amplification factors of the fittest phenotypes 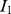. Concretely: (A) 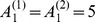; (B) 
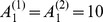
; (C) 
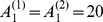
 and (D) 
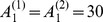
. The rest of the amplification factors are: 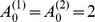 and 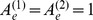 and the mutation rates of 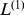 and 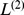 are 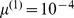 and 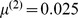, respectively. As in the previous figure, for each experimental setup 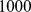 runs were performed for 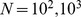 and 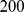 runs for larger populations sizes.

To further investigate how the probability of fixation depends on the initial conditions and the value of the highest peak (largest amplification factor) 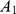, we carry out a serial of experiments whose results are summarized in Fig. 8. In all the simulations the mutation rates for both lineages are fixed: 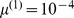 and 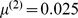 for 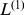 and 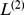, respectively. In the deterministic approach, since the selective value of the fittest phenotype of each lineage verifies 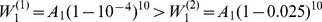 then, the lineage 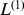 is selected. As can be seen, this is not the case when the size of the population is not high enough to reach the deterministic limit. In fact, even for very large populations sizes (i.e. 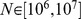), the fixation of 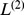 reaches 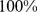 of the simulations. For 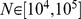 a non-null probability of fixation of 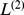 exists that, for a given population size and initial conditions, tends to increase with the amplification factor 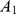. However, for smaller population sizes the internal noise is so high that the fixation of 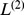 is very low. Note that, even when the initial condition of 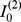 is low a high probability of fixation still exists for population sizes in the interval 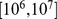.

In summary, all these results confirm that for population sizes which are high, but not high enough to reach the deterministic limit, the lineage with the largest value of the mutation rate (the mutator lineage) can take over the whole population. This is a consequence of arriving first to the fittest phenotype which, by natural selection, displaces the less fit sequences of the other lineage. The important fact is that, a priori, the fittest phenotype, that belongs to the low mutator lineage, is never reached. The question arises as to whether an optimal mutation rate exists that, for a given population size, optimizes the probability of fixation. This question is addressed next by studying the time evolution of a finite population formed by five lineages with different mutation rates.

As in the previous simulations, an initial population divided equally among five lineages with different mutation rates evolves over time until the stationary state, i.e. the selection of one of the lineages, is achieved. Initially, only sequences 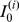 exist. In all lineages the amplification factor of the error tail is 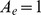. The mutation rates of each lineage take the values already highlighted in Fig. 4, concretely: 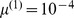, 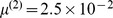, 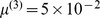, 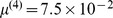 and 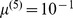. The amplification factors of the fittest sequence in each lineage are equal and is varied in the simulations (see Table 2). The population size ranges from 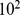 to 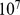. As before, the results shown in Table 2 are obtained from 1000 simulations for 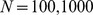 and 200 simulations for larger population sizes. As can be seen in this table, for all values of 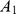 and population sizes in the interval 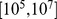 the lineage 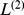 is selected. Importantly, this lineage has the mutation rate that yields the lowest characteristic time of its fittest sequence, i.e. 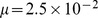. For 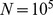 and amplification factor 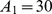, the lineage 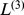 presents similar percentages of selection. On the contrary, for low population sizes (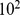 and 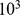) and values of the amplification factor 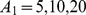, the lineage 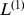 with the lower mutation rate and a large selective value is mostly selected. For the intermediate size 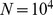 none of the lineages have a clear selective advantage, which is likely due to the fact that the selection pressure and time evolution are almost compensated. In any case, these results suggest that, at least for an intermediate range of the population sizes 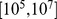, the mutation rate that provides the minimum 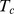 is highly correlated with the probability of survival under a selective pressure.

**Table 2:** Percentage of fixation of each lineage during the stochastic competition of five lineages.

*A*_1_	2*N	Mutation rate of each lineage				
		10^−4^	2.5×10^−2^	5×10^−2^	7.5±10^−2^	10^−1^
5	10^2^	97.8	0.9	0.8	0.5	0
	10^3^	84.5	5.6	6.6	3.3	0
	10^4^	16	43.5	27.5	13	0
	10^5^	0	99.5	0.5	0	0
	10^6^	0	100	0	0	0
	10^7^	0	100	0	0	0
10	10^2^	94.8	1.5	1.3	0.8	1.6
	10^3^	64.5	5.9	10.6	11.2	7.8
	10^4^	4	31.5	47	14.5	3
	10^5^	0	95	5	0	0
	10^6^	0	100	0	0	0
	10^7^	0	100	0	0	0
20	10^2^	93	1.8	1.6	1.5	2.1
	10^3^	57.5	5.9	13	12.2	11.4
	10^4^	0	21	33	33.5	12.5
	10^5^	0	66.5	32	1.5	0
	10^6^	0	100	0	0	0
	10^7^	0	100	0	0	0
30	10^2^	93.6	1.7	1.5	0.9	2.3
	10^3^	54.5	6.8	12.3	13.4	13
	10^4^	0.5	13.5	33.5	26	26.5
	10^5^	0	48.5	47.5	4	0
	10^6^	0	98.5	1.5	0	0
	10^7^	0	100	0	0	0

Percentage of fixation of each of the five lineages with different mutation rates in terms of the population size (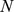) and the amplification factor of the fittest phenotype (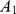). As before, 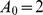 and 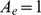 in all the lineages. For each experimental setup 1000 runs were performed for 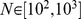, and 200 runs for 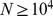.

## Discussion

The validity of the infinite size approximation, i.e. the deterministic approach, depends very much on the problem under study. For instance, finite size effects do not seem to be particularly relevant for the formation of a quasispecies around a wild type phenotype [25]. In contrast, this limit is scarcely valid for quantifying the evolutionary time of populations of sequences or lineages of sequences. It turns out that for small population sizes evolution can be practically impeded due to the huge increase of the searching time, i.e. the time needed to find a better phenotype. This fact can change the fate of evolution as deduced from the deterministic description. In this paper, we have explored the evolutionary time in finite populations using a simple model of quasispecies lineages that evolve in a two-peak landscape. Importantly, the finite size effects are so drastic that the deterministic limit cannot be applied to predict the evolution outcome. The question of how large the population must be to assure the deterministic limit is, in our opinion, of great interest. Nonetheless, as has been pointed out above, it depends on the intrinsic characteristics of the problem, in particular, on the fitness landscape, the mutation rates and initial conditions. Furthermore, there is no clear way of determining the dependence of the internal fluctuations on these factors, and its exploration using computer simulations is almost impossible due to the large size of the system.

The mutation rate is an essential parameter to determine the time taken to reach the fittest sequence from the master one and to stabilize. In the deterministic limit, this time can be estimated by the characteristic time [14]. An analog to this time can be used to estimate an evolutionary time in finite size populations [16]. The question arises as to what extent the characteristic time associated to a lineage (e.g. that of its fittest phenotype) is responsible for its survival. In other words, whether lineages with low characteristic time have a larger probability of being selected by natural selection in a finite population. It must be stressed at this point that, in the deterministic limit and above the error threshold, the only factor that determines the final outcome of the evolutionary process is the selective value of the phenotypes, independently of their characteristic time. It turns out that, as Fig. 2B and Fig. 6B depict, the error tail concentration is very low in the transition from the master sequence to the fittest one, and then internal noise caused by the finite size of the population becomes relevant. The major consequence is that the fate of evolution, as predicted by the deterministic model, can be drastically modified when dealing with finite size populations. As presented in the Results, this disparity is especially important when independent linages are competing in a constrained finite population. This has already been obtained in [12], [13] for a system formed by two independent populations (lineages) of error-prone replicators in a different fitness landscape. Here, we have interpreted this stochastic outcome from the characteristic time of the lineage (measured from the characteristic time of their fittest phenotypes). The lineage that arrives first to its fittest phenotype is able to displace the best adapted one, as deduced from a deterministic approach, only in the case of finite size populations.

As has been stated above, the assumption that lineages are mutationally isolated is valid when sequence evolution occurs without a significative modification of their mutation rate. This hypothesis allows a complete computational treatment of the population dynamics even when it is formed by five lineages. When longer time scales are considered, lineages can then be mutationally connected and hence, the mutation rate can evolve. For instance, it could be assumed that some digits of the sequence (a *locus*) codify the mutation rate of the whole sequence. Under this assumption, a mutator phenotype could be fixed due to its better selective value, but later the population would evolve again towards a phenotype with a lower mutation rate. It is likely that a phenomenon of transient “switch” in the mutation rate would occur, similar to that described by [26]. However, this dynamics could be radically different if more complex fitness landscapes, that might be not only rugged but also dynamic, i.e. that change over time, are taken into account. Obviously, this raises the question as to what extent the results obtained from these models are applicable to real fitness landscape, e.g that of viruses. Recent papers have provided new experimental data and confirm that they are in general more rugged, as expected, and with a high level of neutrality (see [27] and references therein). Neutrality is already present in the two peak landscape and, indeed, causes the drastic rise of the characteristic time to achieve the fittest peak observed for finite size populations. In addition, this fitness degeneracy is partly responsible for the strong discrepancy with the deterministic outcome. The effect of ruggedness on the characteristic time has already been studied in a previous paper [15]. In that paper we studied the characteristic time of a population of replicators in more rugged landscapes, namely multiplicative with two peaks, binary rugged and Kauffman-NK landscapes, and showed that it presents a similar dependence on the mutation rate. Therefore, although real fitness landscapes are essentially more complex and differ globally from the double peak landscape model assumed in our study, the results derived under this assumption are of great interest from a local perspective, i.e a dynamics restricted to successive moves from one master phenotype to another master in its neighborhood in a relatively short time scale.

The matter of whether the high value of the mutation rates of viruses results from natural selection is still under debate. Two main explanatory lines have been stated. On the one hand, natural selection would foster high mutation rates because they confer a large adaptability to environmental changes. A lower bound to the mutation rate would appear to maintain the information of the quasispecies, the error threshold. So, according to this line of reasoning, an optimal mutation rate will exist placed just above this error threshold [28]. On the other hand, an alternative explanation comes from the evidence that evolution is only acting instantaneously and on finite size populations. Therefore, natural selection would not be able to cross extended valleys between fitness peaks, mainly caused by the accumulation of deleterious mutations [29]. From this perspective, a high mutation rate would appear as a side effect of selection for high replicative rates [30], [31] (a classical example of “selection for” instead of “selection of” [32]).

This paper has shown that both explanations are not mutually contradictory. On the contrary, they explain two different manifestations of natural selection acting on populations with different sizes. The fixation of the mutation rates during evolution depends strongly on the population size and it is highly likely that the direction of adaptation might have changed repeatedly according to the selective pressures that operate at each moment. When the population size is low, the characteristic time is high and the deleterious effect of the mutation rate causes the disappearance of the mutator lineage. For medium size populations, the adaptive capacity of the mutator lineage, reflected in its ability to arrive first to its fittest phenotypes, overcomes the deleterious effects and allows its selection over the entire population. For even larger population sizes, in the deterministic limit, the greater adaptability of the mutator lineage is no longer enough to displace the non-mutator lineage, as this has similar potential to achieving its fittest phenotype before disappearing, and then becoming fixed (to the detriment of the mutator lineage).

A question that immediately arises from this discussion is whether the selection of mutator lineages is a consequence of a hitchhiking phenomenon, i.e. the selection of mutator alleles because they are linked to other advantageous alleles that are effectively selected by natural selection [24], [33]–[37]. In light of our results, these mutator alleles are selected because of their selective advantage provided by a shorter evolutionary time. For intermediate population sizes, a phenotype that belongs to a lineage that has the shortest evolutionary time enhances its probability of being selected. This phenotype gets an advantage not only by lifting a lineage but by riding the fastest one.

## Methods

In some cases it is reasonable to describe the time evolution of a population formed by several phenotypes in terms of continuos variables, such as molar fractions. In the homogeneous case, the dynamics of each variable is usually described in terms of Ordinary Differential Equations. If the system exhibits an asymptotic behavior, all molar fractions approach their equilibrium values and, by definition, the time they take to achieve this state is infinite. However, this mathematical information has low value in many practical problems where a stationary regime is approximately reached in a finite time. Many different methods have been suggested to get an estimation of the scale of this intrinsic system dynamics. We recently presented in [15] the characteristic time of a continuos variable as a way of overcoming important deficiencies in previous definitions, particularly for non-linear systems, by taking into account the whole path from the initial condition to the final state of a given trajectory. As discussed in that paper, this characteristic time can be interpreted as: (i) specifically for linear system, as a weighted average of the inverse of the system eigenvalues, (ii) the hypothetical time at which the whole transition occurs and (iii) the mean time of the transition. More recently, this concept has also been applied to estimate the characteristic time of stochastic population systems where internal noise is considered [16].

The ODE systems that describe the time evolution of the population in the deterministic approximation that assumes an infinite size Eq. (3) are solved numerically using a Runge-Kutta scheme implemented under the standard software MATLAB. Numerical integration is stopped when the molar fraction of the fittest phenotype 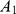 at successive steps differs less than 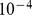 during at least 200 consecutive steps. It is assumed that initially only phenotypes 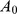 exist in the population. When more than one lineages is present, the same proportion of phenotypes 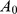 of each lineages is initially supposed. The trajectories of the phenotype with the largest amplification factor are then used to compute the characteristic time as described in Llorens *et al.* [14]. To be precise, if 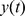 is a monotonous trajectory of the dynamical system with initial condition 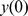 and equilibrium point then its characteristic time reads:
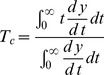
(6)


For finite size populations we have used a well-known stochastic approach, the so-called Gillespie's algorithm, to simulate the time evolution of the number of sequences of the possible phenotypes that can be formed in the system. The Gillespie's algorithm provides an exact simulation of the time evolution of the number of genomes of different phenotypes in a finite population [21]. The algorithm was implemented in C. To generate pseudorandom numbers we apply the Mersenne twister method [38]. To compute the characteristic time of the fittest sequence, the program controls the asymptotic phase of the simulations and determines the first time the population of phenotypes 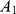 becomes larger than that of phenotypes 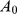, that is denoted as 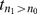. Here 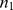 and 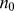 represent the number of genomes with phenotypes 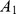 and 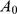, respectively. The final time of each simulation is taken as:
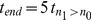
(7)


In so doing, we are assuring that the number of genomes of phenotype 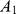 is already in its asymptotic phase and then, its mean value is close to its steady state. A stochastic characteristic time is computed in a MATLAB framework according to the procedure previously described in [16]. Essentially, we approximate the stochastic realization by a monotonously increasing curve that converges to the mean value of the last 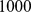 values of 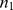. This resulting continuous curve is then used to estimate the characteristic time of a single simulation by means of the formula Eq. (6). Finally, the average characteristic time is computed from 200 simulations. In addition, the standard deviation is also determined for each experimental setup. In the experiments that involve more than one lineage, a checking step for lineage disappearance is included in the program. In the case of two lineages, the simulation is stopped when all phenotypes that form a lineage have died off. When five lineages are competing, the simulation ends when four lineages disappear.
